# Supplementary material for: Functional control of electrophysiological network architecture using direct neurostimulation in humans
Source: Netw Neurosci. 2019 Jul 1;3(3):848–77. doi: 10.1162/netn_a_00089 (PMC6663306; doi:10.1162/netn_a_00089)
Supplement: Supplementary file 1 [file netn-03-848-s001.pdf]

Khambhati, A.N., Kahn, A.E., Costantini, J., Ezzyat, Y., Solomon, E.A., Gross, R.E., Jobst, B.C., Sheth, S.A., Zaghloul, K.A., Worrell, G., Seger, S., Lega, B.C., Weiss, S., Sperling, M.R., & Gorniak, R. (2019). Supporting information for "Functional control of electrophysiological network architecture using direct neurostimulation in humans." *Network Neuroscience*, *3*(3), 848–877. https://doi.org/10.1162/netn_a_00089

SUPPLEMENTARY INFORMATION: Functional control of electrophysiological network architecture using direct neurostimulation in humans

Ankit N. Khambhati[a], Ari E. Kahn[a,c,i], Julia Costantini[a], Youssef Ezzyat[d], Ethan A. Solomon[a], Robert E. Gross[j], Barbara C. Jobst[k], Sameer A. Sheth[l], Kareem A. Zaghloul[m], Gregory Worrell[n], Sarah Seger[o], Bradley C. Lega[o], Shennan Weiss[p], Michael R. Sperling[p], Richard Gorniak[q], Sandhitsu R. Das[e], Joel M. Stein[h], Daniel S. Rizzuto[d], Michael J. Kahana[d], Timothy H. Lucas[g], Kathryn A. Davis[e], Joseph I. Tracy[p], Danielle S. Bassett[a,b,e,f,1]

[a]*Department of Bioengineering, University of Pennsylvania, Philadelphia, PA 19104, USA*
[b]*Department of Electrical & Systems Engineering, University of Pennsylvania, Philadelphia, PA 19104, USA*
[c]*Department of Neuroscience, University of Pennsylvania, Philadelphia, PA 19104, USA*
[d]*Department of Psychology, University of Pennsylvania, Philadelphia, PA 19104, USA*
[e]*Department of Neurology, Hospital of the University of Pennsylvania, Philadelphia, PA 19104, USA*
[f]*Department of Physics & Astronomy, University of Pennsylvania, Philadelphia, PA 19104, USA*
[g]*Department of Neurosurgery, Hospital of the University of Pennsylvania, Philadelphia, PA 19104, USA*
[h]*Department of Radiology, Hospital of the University of Pennsylvania, Philadelphia, PA 19104, USA*
[i]*Human Research and Engineering Directorate, U.S. Army Research Laboratory, Aberdeen, MD 21001 USA*
[j]*Department of Neurosurgery, Emory University Hospital, Atlanta, GA 30322, USA*
[k]*Department of Neurology, Dartmouth-Hitchcock Medical Center, Lebanon, NH 03756, USA*
[l]*Department of Neurosurgery, Baylor College of Medicine, Houston, TX 77030, USA*
[m]*Surgical Neurology Branch, National Institutes of Health, Bethesda, MD 20814, USA*
[n]*Department of Neurology, Mayo Clinic, Rochester, MN 55905, USA*
[o]*Department of Neurosurgery, University of Texas, Southwestern Medical Center, Dallas, TX 75390, USA*
[p]*Department of Neurology, Thomas Jefferson University Hospital, Philadelphia, PA 19107, USA*
[q]*Department of Radiology, Thomas Jefferson University Hospital, Philadelphia, PA 19107, USA*

---

[1]To whom correspondence should be addressed: dsb@seas.upenn.edu

Supplementary Data

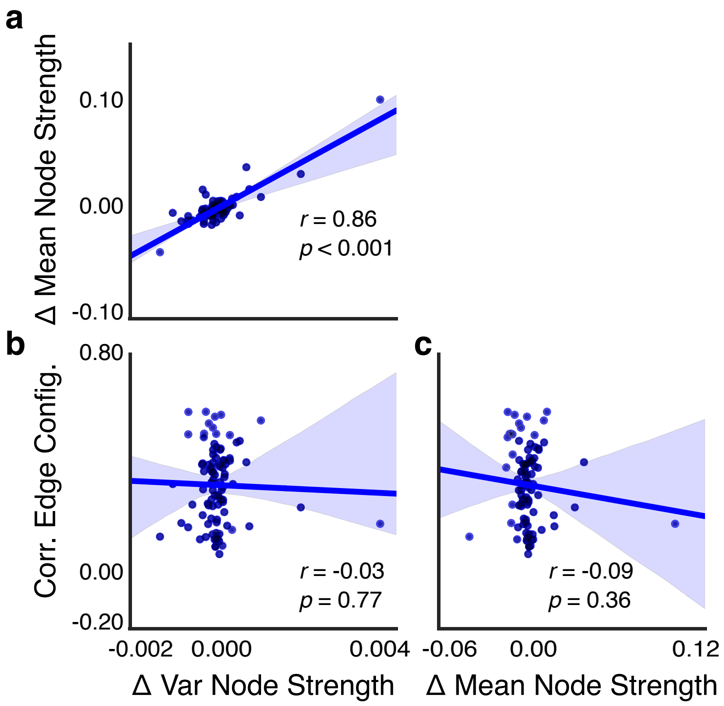

Figure 1-1: Relationship between measurements of functional network reconfiguration. In this study, we compute the following three measurements of functional network reconfiguration: (i) change in the mean of node strengths, (ii) change in the variance of node strengths, and (iii) change in the edge configuration of functional interactions. To quantify the extent that these three measurements capture similar features of topological change in the network, we compute the correlation between each pair of measurements over stimulation sessions across subjects. (**a**) We find that the change in the variance of node strengths due to stimulation is significantly correlated with the change in mean of node strengths (Pearson's $r(246) = 0.86$, $p < 0.001$, Bonferroni corrected). This result implies that as nodes exhibit more coherent interactions with other nodes in the network, the network exhibits a more heterogeneous profile of node strengths – where nodes may exhibit, both, stronger and weaker coherences with other nodes in the network. (**b**) We find that the change in the variance of node strengths due to stimulation exhibits low, non-significant correlation with the change in edge configuration (Pearson's $r(246) = -0.03$, $p = 0.77$, Bonferroni corrected). This result implies that stimulation may drive reconfiguration of the functional interactions in the network independent of the change in heterogeneity of the set of node strengths in the network. (**c**) We find that the change in the mean of node strengths due to stimulation exhibits low, non-significant correlation with the change in edge configuration (Pearson's $r(246) = -0.09$, $p = 0.36$, Bonferroni corrected). This result implies that stimulation may drive reconfiguration of the functional interactions in the network independent of the change in the average strength of coherent interactions at the scale of network nodes. Each observation is a stimulation session of a single subject.

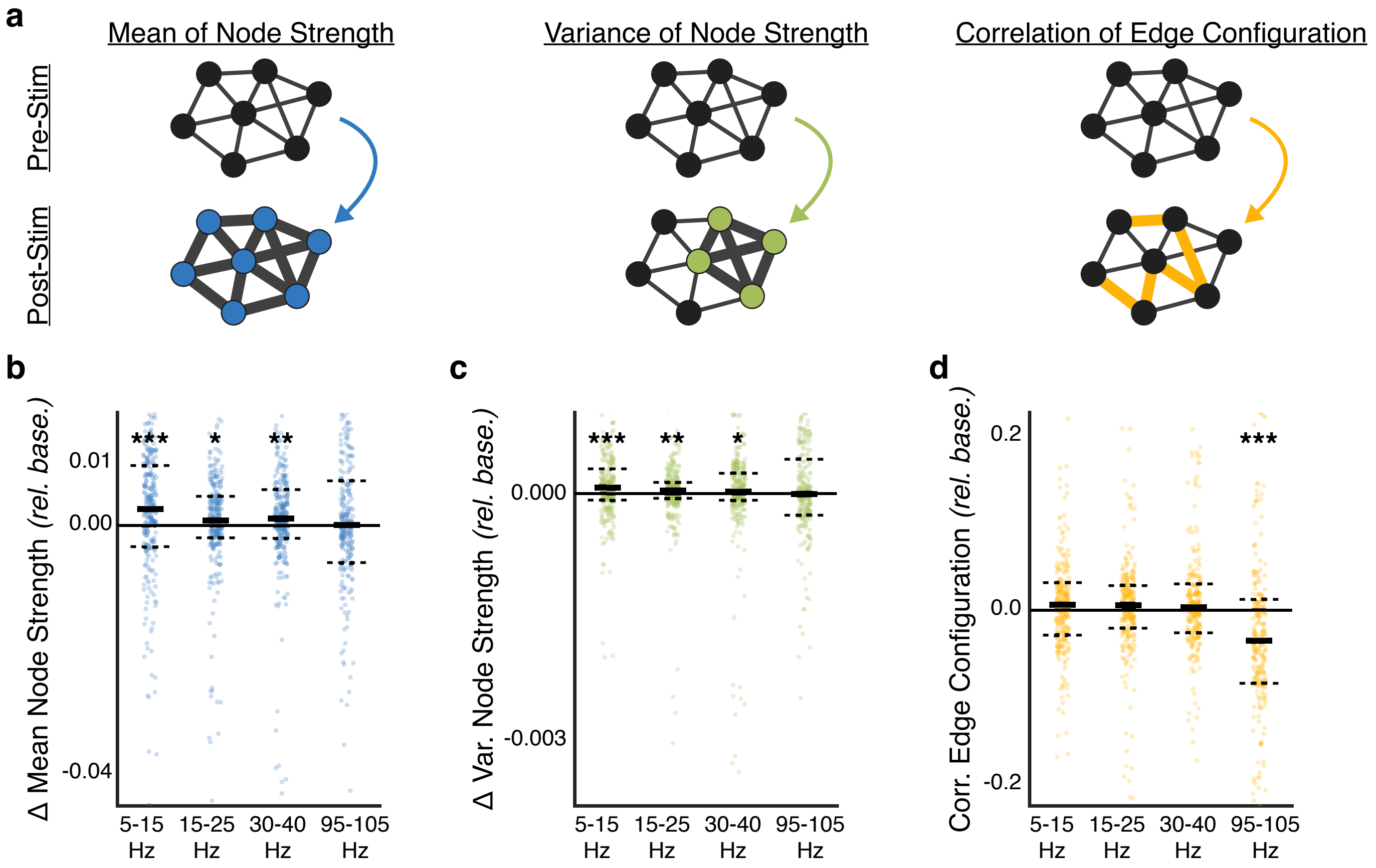

Figure 2-1: Control of frequency-specific functional network topology (600ms post-stimulation). (**a**) Does stimulation induce network reconfiguration at the scale of network nodes or at the scale of network edges? Shown here are different forms of network reconfiguration: two forms at the node scale and one form at the edge scale. At the node scale, stimulation may increase or decrease the overall functional interactions of a node with other nodes in the network, resulting in a change in the mean of node strengths and/or a change in the variance, or heterogeneity, of node strengths in the network. At the edge scale, stimulation may alter the configurational pattern of functional interactions underlying functional network topology. We measure edge scale change by computing a configuration similarity metric of the pattern of network coherences between the pre-stim trial and the post-stim trial; values near 1 (or 0) imply a lesser (or greater) change in network configuration. (**b**) Difference in the change in mean of node strengths between stim epochs and baseline epochs. Change in the mean of node strengths is significantly greater during stimulation epochs than during baseline epochs in the alpha/theta band ($p < 0.001$, corrected), in the beta band ($p < 0.05$, corrected), and in the low gamma band ($p < 0.01$, corrected). (**c**) Difference in the change in variance of node strengths between stim epochs and baseline epochs. Change in the variance of node strengths is significantly greater during stimulation epochs than baseline epochs in the alpha/theta band ($p < 0.001$, corrected), in the beta band ($p < 0.01$, corrected), and in the low gamma band ($p < 0.05$, corrected). (**d**) Difference in the configuration similarity of network edges between stim epochs and baseline epochs. Reconfiguration of functional interactions is significantly greater during stimulation epochs than during baseline epochs in the high gamma band ($p < 0.001$, corrected). Each observation is the average across epochs within a stimulation session of a single subject. Solid lines represent the median, and dashed lines represent the first and third quantiles. $*p < 0.05$, $**p < 0.01$, $***p < 0.001$.

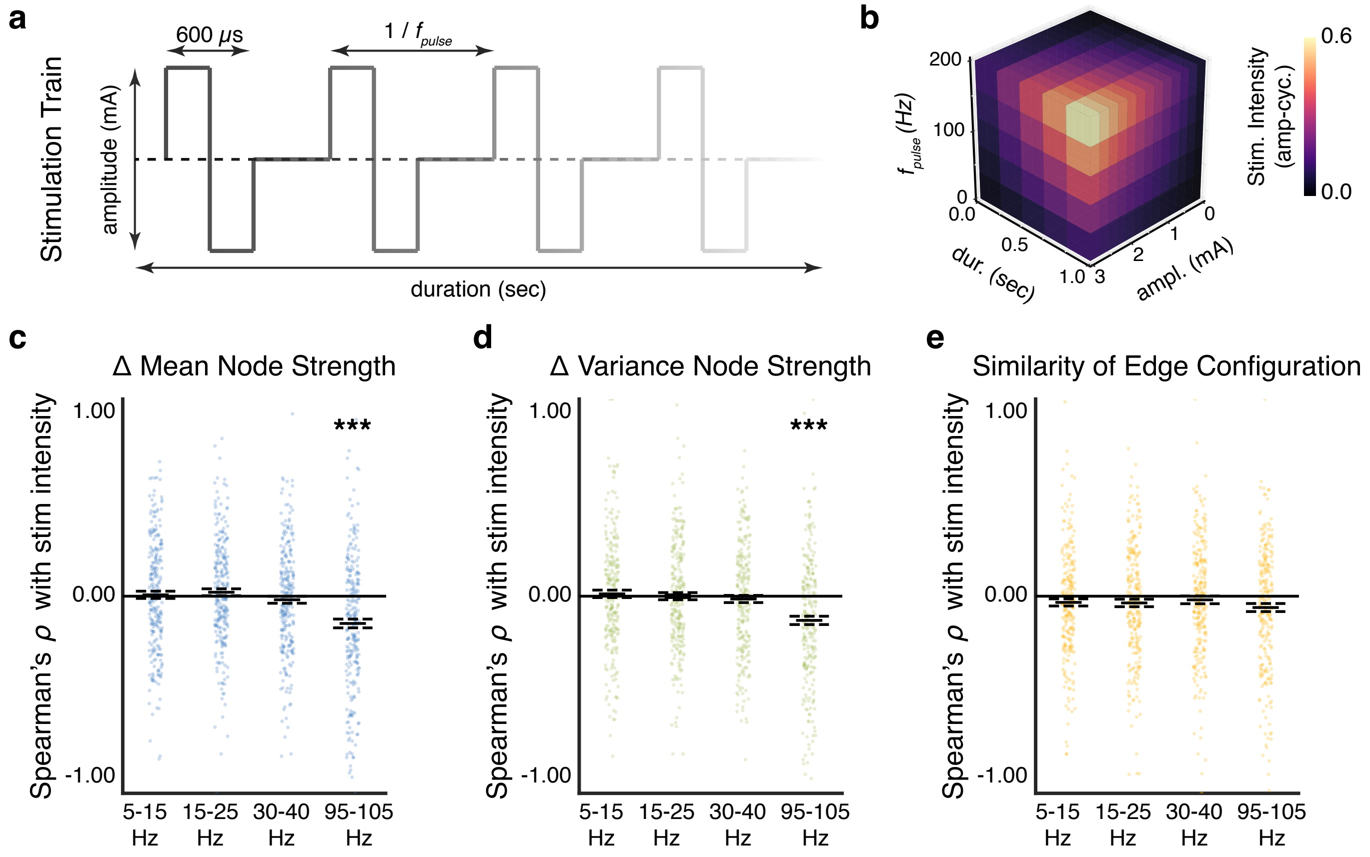

Figure 3-1: Dose-dependent response of network reconfiguration to stimulation (600ms post-stimulation). (**a**) To examine the effect of stimulation intensity on network reconfiguration, we vary the amplitude, pulse frequency, and duration of the square-wave input. (**b**) We quantify the total input intensity delivered during a stimulation trial as the product between the amplitude, pulse frequency, and duration. Here, we show the three-dimensional plane of input parameters that contribute to the overall stimulation intensity. (**c**) Distribution of correlations between the stimulation intensity and the change in mean of node strengths. Correlations are significantly negative in the high gamma band ($p < 0.001$, corrected). (**d**) Distribution of correlations between the stimulation intensity and the change in variance of node strengths. Correlations are significantly negative in the high gamma band ($p < 0.001$, corrected). (**e**) Distribution of correlations between the stimulation intensity and the configuration similarity. Correlations are non-significant across all frequency bands. Each observation is the correlation across epochs within a stimulation session of a single subject. Solid lines represent the mean, and dashed lines represent standard error of the mean. *$p < 0.05$, **$p < 0.01$, ***$p < 0.001$.

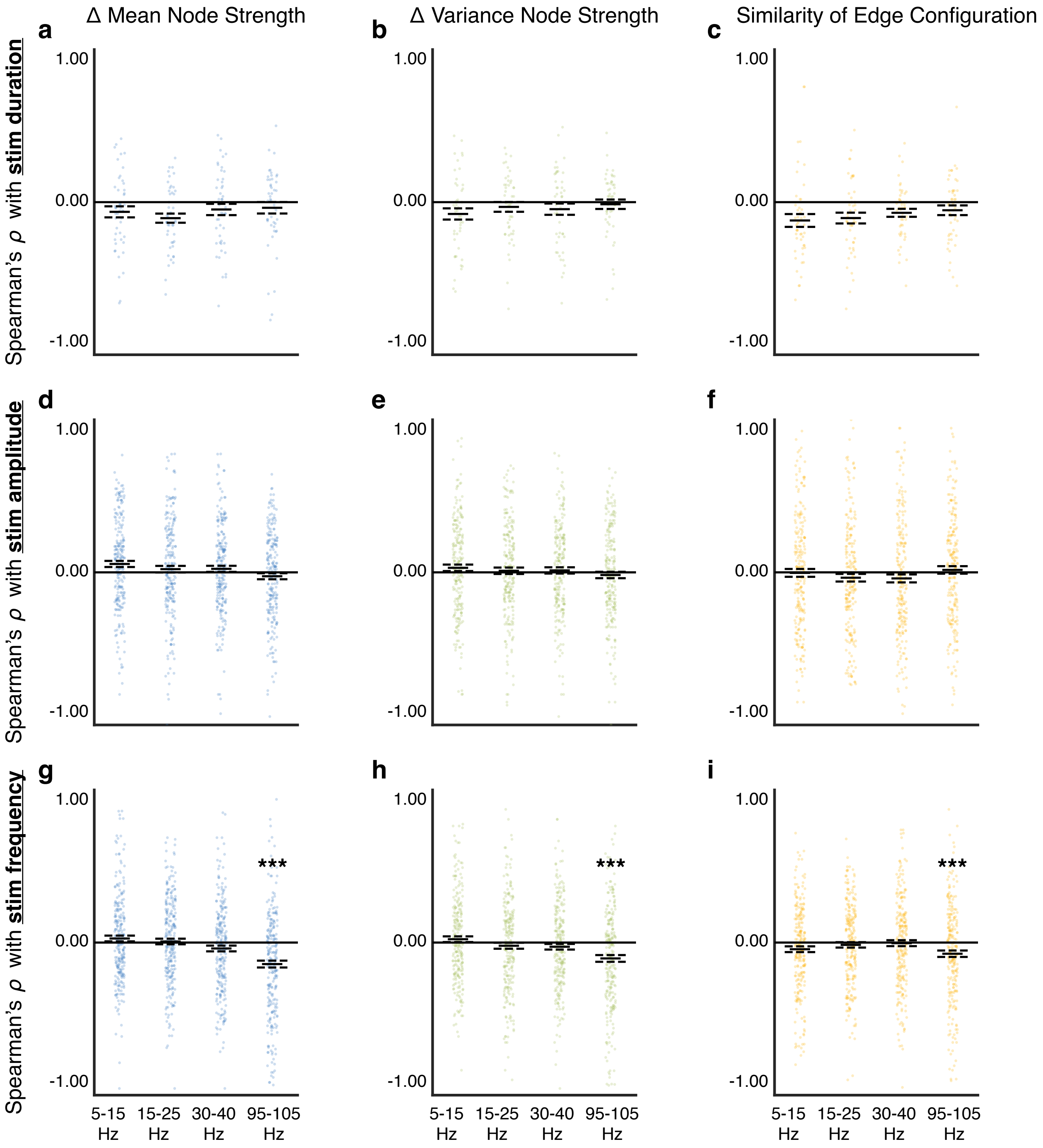

Figure 3-2: Dose-dependent response of network reconfiguration to each dimension of stimulation parameter space. We first computed the Spearman correlation between each parameter dimension (rows of subplots) and each network measure (columns of subplots) over stimulation trials for each subject. We next used a one-sample *t*-test and Bonferroni correction for multiple comparisons testing to assess whether stimulation parameter changes along a single dimension reliably altered a measure of network topology across subjects. We found no significant effect of stimulation duration (**a-c**) and stimulation amplitude (**d-f**) on the three measures of network reconfiguration in any of the four frequency bands. In the high gamma band, we found that slower stimulation frequencies tended to increase the strength of network nodes (**g-h**) and faster stimulation frequencies tended to disrupt the strength of network nodes and drive greater edge-level reconfiguration (**i**). These findings imply that by varying pulse frequency, neurostimulation may be used to reinforce or disrupt node-level topology and drive edge-level reconfiguration in human brain networks. Solid lines represent the mean, and dashed lines represent standard error of the mean. $*p < 0.05$, $**p < 0.01$, $***p < 0.001$.

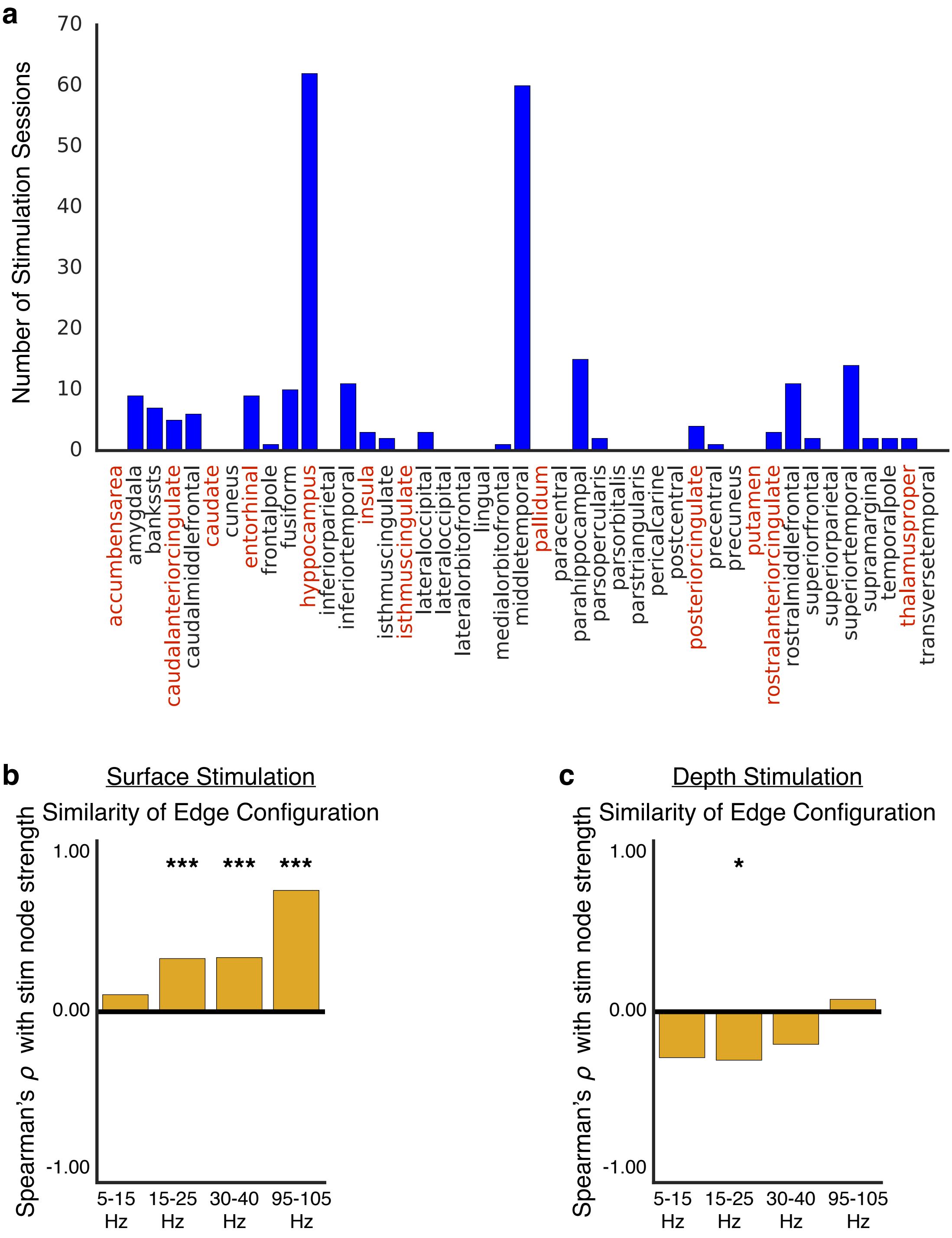

Figure 4-1: Regional effects of stimulation on network reconfiguration (300ms post-stimulation). (**a**) Distribution of 248 stimulation locations across brain regions defined by the Lausanne atlas included in the Connectome Mapping Toolkit [1]. Regions defined as depth areas are labeled in red. (**b**) Correlation between stimulation node strength and configuration similarity amongst surface stimulation locations. Correlations are significantly positive in the beta band ($p < 0.001$, corrected), in the low gamma band ($p < 0.001$, corrected), and in the high gamma band ($p < 0.001$, corrected). (**c**) Correlation between stimulation node strength and configuration similarity amongst depth stimulation locations. Correlations are significantly negative in the beta band ($p < 0.05$, corrected). These results suggest that stimulation of stronger functional hubs at the cortical surface may drive network reconfiguration of higher frequencies, while stimulation of stronger functional hubs in the depth may drive drive network reconfiguration of lower frequencies. Correlations are computed over stimulation sessions across subjects. $*p < 0.05$, $**p < 0.01$, $***p < 0.001$.

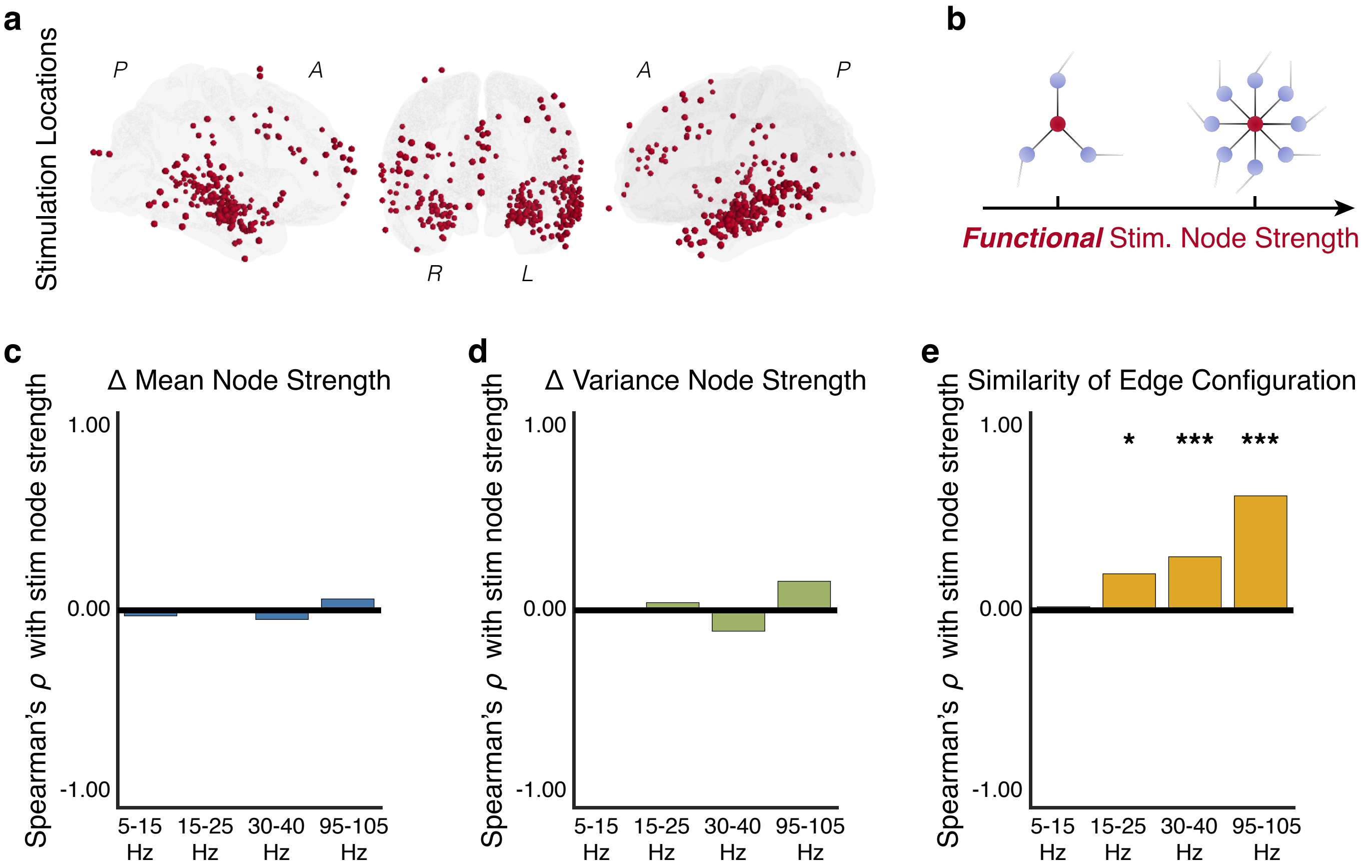

Figure 4-2: Functional hubs constrain topological response to stimulation (600ms post-stimulation). (**a**) Distribution of 248 stimulation locations sampled across 94 subjects. (**b**) To examine the effect of stimulation location on the reconfiguration of functional network topology, we measure the node strength of the stimulation region during the baseline epoch – before any stimulation is delivered – for each coherence frequency band. Intuitively, nodes with low strength (*left*) tend to be functionally isolated and exhibit weak coherence with the other nodes in the network, while nodes with high strength (*right*) tend to be functional hubs and exhibit strong coherence with the other nodes in the network. We expect that stimulation of strong functional hubs will lead to a homogenous change in network topology, and stimulation of weak functional hubs will lead to a heterogenous change in network topology. (**c**) Correlation between stimulation node strength and change in mean of node strengths. We find no significant relationship between stimulation node strength and change in mean of node strengths in any frequency band. (**d**) Correlation between stimulation node strength and change in variance of node strengths. We find no significant relationship between stimulation node strength and change in variance of node strengths. (**e**) Correlation between stimulation node strength and configuration similarity. Correlations are significantly positive in the beta band ($p < 0.05$, corrected), in the low gamma band ($p < 0.001$, corrected), and in the high gamma band ($p < 0.001$, corrected). Correlations are computed over stimulation sessions across subjects. *$p < 0.05$, **$p < 0.01$, ***$p < 0.001$.

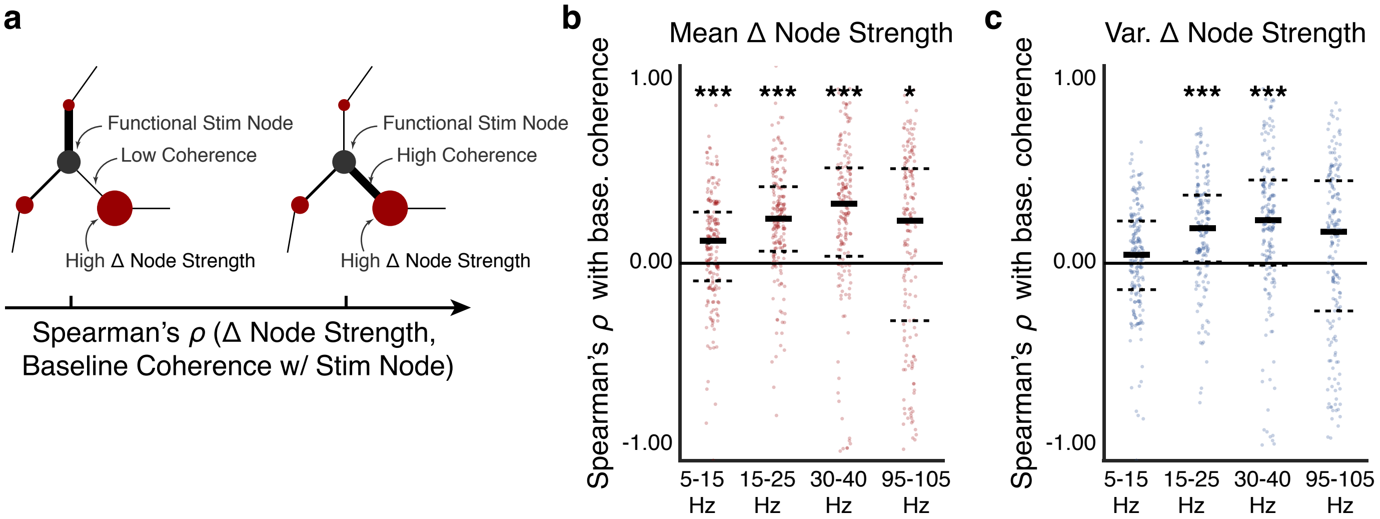

Figure 5-1: Predicting downstream modulation of regional coherence (600ms post-stimulation). (**a**) We hypothesize that the baseline strength of coherent interactions between the stimulation node (red) and network nodes away from the stimulation node (black) predicts the likelihood that these downstream nodes will be evoked due to stimulation. Intuitively, a weak baseline coherence between the stimulation node and a downstream network node is less likely to modulate the mean coherence of the downstream node (*left*), and a strong baseline coherence between the stimulation node and a downstream network node is more likely to modulate the mean coherence of the downstream node (*right*). To test this hypothesis, we first quantify the magnitude change in node strength within each stimulation session. We next compute Spearman's ρ correlation between the mean (and variance) of change in downstream node strength across stimulation trials and the baseline coherence between the stimulation node and the downstream nodes. (**b**) Distribution of correlations between the mean of change in downstream node strength and the baseline coherence between the stimulation node and the downstream nodes, for each of the four coherence frequency bands. We find a significantly positive correlation in the alpha/theta band ($p < 0.001$, corrected), in the beta band ($p < 0.001$, corrected), in the low gamma band ($p < 0.001$, corrected), and in the high gamma band ($p < 0.05$, corrected). (**c**) Distribution of correlations between the variance of change in downstream node strength and the baseline coherence between the stimulation node and the downstream nodes. We find a significantly positive correlation in the beta band ($p < 0.001$, corrected) and in the low gamma band ($p < 0.001$, corrected). These results suggest that baseline functional network topology involving the stimulation node predicts the flexibility with which a downstream node may alter its interactions with other nodes in the network. Each observation is the correlation within a stimulation session of a single subject. Solid lines represent the median, and dashed lines represent the first and third quantiles. $*p < 0.05$, $**p < 0.01$, $***p < 0.001$.

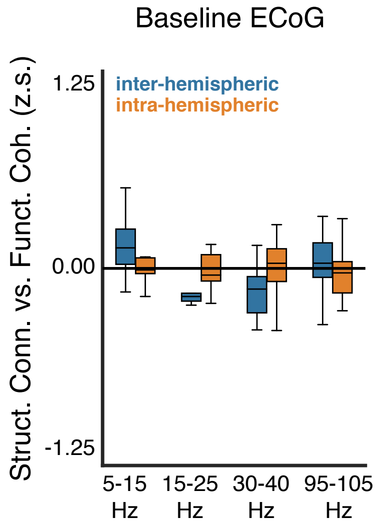

Figure 6-1: Correlation between Structural Connectivity and Baseline Coherence (N = 234 ROIs). Each observation is a Fisher Z-transformed correlation between structural connectivity from DTI and baseline coherence from ECoG in a single subject based on the coarse Lausanne parcellation of the brain. Boxplots represent the distributions of correlation values across subjects, subdivided by inter-hemispheric and intra-hemispheric connections and coherence frequency band. In contrast with the finer-scale Lausanne parcellation (N = 1015 ROIs), we find no significant relationship between structural connectivity and baseline functional connectivity in any of the four frequency bands ($p \geq 0.05$). These results imply that white matter fiber connectivity constrains ECoG functional connectivity at a scale 1-2 cm. At larger spatial scales, LFP-based functional interactions explainable by white matter connections may become obscured by smoothing neural dynamics over brain tissue 6 cm^3 in volume.

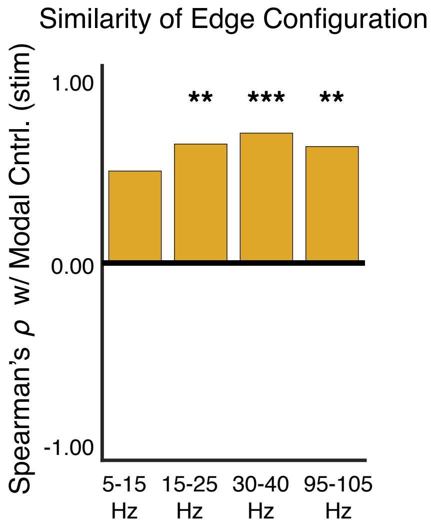

Figure 6-2: Using neurostimulation to bridge structure, function, and behavior (600ms post-stimulation). Correlation between modal controllability of the stimulated brain region and average functional configuration similarity across stimulation sessions. We find a significant positive correlation in the beta band ($p < 0.01$, corrected), in the low gamma band ($p < 0.001$, corrected), and in the high gamma band ($p < 0.01$, corrected). $*p < 0.05$, $**p < 0.01$, $***p < 0.001$.

References

[1] Cammoun, L., Gigandet, X., Meskaldji, D., Thiran, J. P., Sporns, O., Do, K. Q., Maeder, P., Meuli, R., Hagmann, P., 2012. Mapping the human connectome at multiple scales with diffusion spectrum MRI. Journal of Neuroscience Methods 203 (2), 386–397.
